# Knowledge, Attitude, and Practice of Brazilian Physicians about Immediate Postpartum and Postabortion Intrauterine Device Insertion

**DOI:** 10.1055/s-0043-1772187

**Published:** 2023-10-16

**Authors:** Adalberto Kiochi Aguemi, Maria Regina Torloni, Mirna Namie Okamura, Cristina Aparecida Falbo Guazzelli

**Affiliations:** 1 Women's Health Technical Area, Secretaria Municipal da Saúde de São Paulo, SP, Brazil; 2 Escola Paulista de Medicina, Universidade Federal de São Paulo, SP, Brazil; 3 Coordination of Epidemiology and Information, Secretaria Municipal da Saúde de São Paulo, SP, Brazil

**Keywords:** copper intrauterine devices, postpartum period, spontaneous abortion, induced abortion, health knowledge, attitudes, practice, dispositivos intrauterinos de cobre, período pós-parto, aborto espontâneo, aborto induzido, conhecimentos, atitudes e prática em saúde

## Abstract

**Objective** To assess the knowledge, attitude, and practice of Brazilian physicians about immediate postpartum and postabortion intrauterine device insertion.

**Methods** Cross-sectional online survey involving physicians on duty in public Brazilian hospitals. Participants answered an anonymous questionnaire with close-ended questions to assess their knowledge, attitude, and experience on the immediate postpartum and postabortion insertion of copper intrauterine devices.

**Results** One hundred twenty-seven physicians working in 23 hospitals in the 5 geographic regions of Brazil completed the questionnaire. Most were female (68.5%) and worked in teaching hospitals (95.3%). The mean (standard deviation) knowledge score (0–10 scale) was 5.3 (1.3); only 27.6% of the participants had overall scores ≥ 7.0. Most physicians (73.2%) would insert a postpartum intrauterine device in themselves/family members. About 42% of respondents stated that they had not received any training on postpartum or postabortion intrauterine device insertion. In the past 12 months, 19.7%, 22.8%, and 53.5% of respondents stated they had not inserted any intrauterine device during a cesarean section, immediately after a vaginal delivery, or after an abortion, respectively.

**Conclusion** Most study participants have a positive attitude toward the insertion of intrauterine devices in the immediate postpartum period, but they have limited knowledge about the use of this contraceptive method. A large percentage of respondents did not have previous training on postpartum and postabortion intrauterine device insertion and had not performed any such insertions in the last 12 months. Strategies are needed to improve the knowledge, training, and experience of Brazilian physicians on immediate postpartum and postabortion intrauterine device insertion.

## Resumo

**Objetivo** Avaliar o conhecimento, atitude e prática de médicos brasileiros sobre a inserção de dispositivos intrauterinos no pós-parto e pós-aborto imediatos.

**Métodos** Estudo transversal com inquérito online envolvendo médicos plantonistas de hospitais públicos brasileiros. Os participantes responderam a um questionário anônimo com perguntas fechadas para avaliar seu conhecimento, atitude e experiência sobre a inserção de dispositivos intrauterinos de cobre no pós-parto e pós-aborto imediatos.

**Resultados** Cento e vinte sete médicos de 23 hospitais localizados nas 5 regiões do Brasil preencheram o questionário. A maioria era do sexo feminino (68,5%) e trabalhava em hospitais de ensino (95,3%). O escore médio (desvio padrão) de conhecimento (escala 0–10) foi 5,3 (1,3); apenas 27,6% tiveram escore ≥ 7,0. A maioria (73,2%) faria inserção de dispositivo intrauterino no pós-parto imediato em si mesma/familiares. Cerca de 42% dos participantes declararam não ter recebido nenhum treinamento sobre inserção de dispositivos intrauterinos no pós-parto ou pós-aborto imediatos. Nos últimos 12 meses, 19,7%, 22,8% e 53,5% declararam não ter inserido nenhum dispositivo intrauterino durante uma cesárea, após um parto vaginal ou um aborto, respectivamente.

**Conclusão** A maioria dos participantes tem uma atitude positiva em relação à inserção de dispositivos intrauterinos no pós-parto imediato, porém tem um conhecimento limitado sobre esse método. Uma grande porcentagem dos respondentes não teve treinamento sobre inserção de dispositivos intrauterinos no pós-parto ou pós-aborto imediatos e não fez nenhuma inserção desse tipo nos últimos 12 meses. São necessárias estratégias para melhorar o conhecimento, o treinamento e a experiência dos médicos brasileiros sobre a inserção de dispositivos intrauterinos no pós-parto e pós-aborto imediatos.

## Introduction

Unplanned (unwanted or untimely) pregnancy is a global public health problem that affects especially low- and middle-income country (LMIC) populations. 1 Between 2015 and 2019, there were ∼ 121 million unplanned pregnancies (UPs) annually in the world (64 UPs/1,000 women of reproductive age). Over half of these pregnancies ended in induced abortions, exposing nearly 73 million women per year to the risks associated with this procedure, often performed under inadequate conditions because they are illegal in many countries. 1 2 In Brazil, nearly 55% of pregnancies are unplanned, and the prevalence of induced abortion is estimated to be 15%. 3 4 Besides the physical and mental consequences to women and their families, UPs also have economic impacts. 5 It is estimated that the total annual costs of UPs in Brazil amount to 4.10 billion Brazilian Reais, of which 4.07 billion (99.2%) are pregnancy and childbirth costs, and 32.8 million (0.8%) are costs related to miscarriages or abortions. 6

Lack of access to effective contraceptive methods, as well as failure in their use, are major contributors to the high rates of UPs. Modern short-acting contraceptives have a smaller impact on reducing UPs than long-acting contraceptives. 7 The copper intrauterine device (IUD) is a safe and inexpensive long-acting reversible contraceptive method with few contraindications. Copper IUDs can be used for up to 10 years and can be inserted in nulliparous women and adolescents. 8 The contraceptive failure rate of copper IUDs (0.8% pregnancy/year in typical use and 0.6% in perfect use) is comparable to the effectiveness of tubal ligation. 9 However, IUDs are still underused, especially in LMICs. In South America, IUDs are used by less than 5% of women, compared with 10 to 35% of women in middle- and high-income countries. 10 11

Intrauterine device insertion in the immediate postpartum period (IPP-IUD) is safe and could increase the use of this method because this is a period when most women are motivated to avoid a new pregnancy. Intrauterine device insertion immediately after placental extraction does not cause additional discomfort, dispenses pregnancy tests, and precludes the need to schedule a postdischarge visit for device insertion. 12 It is important to encourage the use of an effective contraceptive method in the immediate postpartum period because the risk of UP is high in the first months after giving birth since most women will resume sexual activity within weeks of delivery and many will become fertile soon after, especially if they do not breastfeed. 13 14 However, there are several barriers to expand the use of IPP-IUD, including lack of IUDs, cost or reimbursement issues, physicians' lack of training and their concerns about the risk of expulsion, and women's lack of interest for this contraceptive method. 15 The insertion of an intrauterine device immediately after a spontaneous or induced abortion (IPA-IUD) is another window of opportunity to expand the use of this contraceptive method and prevent UPs. 16 The rapid return of fertility immediately after an abortion, coupled with the fact that most women resume sexual activity within the first 2 weeks after early pregnancy loss, underscore the importance of offering an effective contraceptive method immediately after uterine evacuation. 17 18 Barriers to the use of IPA-IUD include factors related to professionals, such as lack of training on insertion in these patients and fear of complications, as well as women's lack of information and fears regarding this method. 19

Knowledge attitude and practice (KAP) studies are important to plan effective interventions because they describe the current knowledge of a population, as well as its attitude and practice on a given topic. 20 There are several KAP studies about IPP-IUD and IPA-IUD insertion involving health professionals in other countries, but we did not identify similar studies in Brazil. 15 21 22 23 24

The main objective of this study was to assess the knowledge, attitude, and practice of physicians working in Brazilian public hospitals about IPP-IUD and IPA-IUD use. The secondary objective was to identify possible barriers to the use of this method in these institutions.

## Methods

This descriptive cross-sectional study was conducted from February to May 2020. The study design foresaw the inclusion of a representative sample of medium size (> 2,000 deliveries/year) Brazilian public, philanthropic, or mixed (private-public) hospitals. We created a list of all eligible institutions for each of the country's five geographic regions from the Ministry of Health's website. 25 Then, for each region, we used an electronically generated random number list (Microsoft Excel software – Microsoft Corp., Redmond, WA, USA) to identify the institutions that would be invited to participate in the study. The total number of institutions selected in each geographic region was proportional to the number of births in that region in 2019, that is, we included more institutions from the Southeast, Northeast, and South regions, and fewer institutions in the North and Midwest regions. Using this random list of institutions, we contacted (by email and telephone) the directors of the selected institutions and invited them to participate in the study. Hospitals were included in the study after the directors accepted the invitation and the study was approved by the local ethics committees. Study participants were physicians who worked as on-duty professionals in the labor and delivery wards in each of the selected institutions. Physicians who were not fluent in Portuguese or who did not deliver babies were considered ineligible. The directors of the participating institutions sent all eligible physicians a standard e-mail (created by the researchers) explaining the purpose of the study and containing the link to an electronic questionnaire. We included in the study all eligible physicians who accepted the invitation and completed the electronic questionnaire (convenience sample).

The questionnaire was developed by the study authors following the methodological recommendations for knowledge and attitude surveys of the World Health Organization (WHO) and based on similar studies conducted in other countries. 15 20 21 22 23 24 The questionnaire was initially tested in a group of 10 on-duty physicians from maternity hospitals not included in the study, modified and retested on another group of 10 on-duty physicians from these same institutions until all questions and answers were clear to all participants. The final version was converted into an electronic questionnaire (Google forms – Google LLC, Mountain View, CA, USA) to be administered online. The questionnaire was anonymous and divided into two parts. The first part collected participants' characteristic. The second part consisted of nine multiple-choice questions to assess physicians' theoretical knowledge (indications, contraindications, risks, complications) about IPP-IUD and IPA-IUD, three questions to assess participants' attitude toward IUD insertion in women managed in different health settings and sectors, four questions about their personal experience and training in postpartum and postabortion insertions, and three questions about possible barriers to the use of these methods in the public hospital where they worked. We used the best available evidence at the time to create the questions and answers. 8 18 26 27

We present the characteristics of the participants, and the results of the knowledge, attitude, and practice questions descriptively (number, percentages, mean, and standard deviation). We converted the scores of the nine knowledge questions to a decimal scale (0 to 10).

The study was approved by the research ethics committee of UNIFESP-EPM (CAAEE 06756219.0.0000.5505) and by the ethics committees of the participating institutions. We obtained informed consent from all participants electronically, before they had access to the anonymous online questionnaire.

## Results

We contacted the 178 randomly-selected hospitals (50% of the 357 eligible institutions), and 23 agreed to participate. The main reason for refusal, according to the directors of the institutions who responded to our contact, was that their physicians were overloaded due to the first wave of the coronavirus disease 2019 (COVID-19) pandemic that was spreading around the country at that time. Because the situation was getting worse over time, and the public health system was collapsing due to the pandemic, and it was impossible to predict the duration of this situation, we decided to end the study 4 months after it started (May 2020). When we ended the study, 127 physicians working in 23 hospitals located in the 5 geographical regions of Brazil had responded to the questionnaire. Most participants were females (68.5%), had a mean age of 40.6 years, had graduated ∼ 15 years earlier, had completed a residency in obstetrics and gynecology (72.4%), and worked in teaching hospitals (95.3%) located in capital cities (84.2%) in the southeast region (51.2%) of Brazil ( Table 1 ).

**Table 1:** Characteristics of 127 physicians on duty in labor and delivery wards of 23 Brazilian public hospitals

Characteristics	n (%)
Sex	
Female	87 (68.5)
Male	40 (31.5)
Age, years	
Minimum–maximum	24–66
Mean (SD)	40.6 (10.4)
Time since graduation, years	
Minimum–maximum	1–42
Mean (SD)	15.4 (10.7)
Highest degree	
PhD	13 (10.2)
Master's degree	14 (11.0)
OB-GYN residency	92 (72.4)
OB-GYN specialist title	8 (6.3)
Number of hospitals where participants work	
1	93 (73.2)
2	14 (11.0)
3 or more	20 (15.8)
Is the participant's institution a teaching hospital?	
Yes	121 (95.3)
No	6 (4.7)
Weekly workload in participant's institution	
< 24 hours	48 ≥37.8)
> 24 hours	79 (62.2)
Geographic location of participant́s institution	
Southeast (12 institutions)	65 (51.2)
Northeast (5 institutions)	29 (22.8)
South (3 institutions)	16 (12.6)
North (2 institutions)	11 (8.7)
Midwest (1 institution)	6 (4.7)
Location of participant́s institution	
State capital city	107 (84.2)
Other cities	20 (15.8)

Abbreviations: OB-GYN, obstetrics and gynecology; SD, standard deviation.

The mean overall score (standard deviation) of the knowledge questions was 5.3 (1.3), ranging from 2.2 to 8.8 (0–10 scale). Only 27.6% ( *n* = 35) of the 127 participants had overall scores ≥ 7. Over ¾ (77.2%) of the physicians overestimated the expulsion rate of IUDs inserted during a cesarean section, most (55.1%) overestimated the risk of expulsion of IUDs inserted immediately after a vaginal birth, and ∼ 61% ( *n* = 77) overestimated the risk of uterine perforation in IUDs inserted after a vaginal birth. On the other hand, most (50.4%) of the participants underestimated the risk of uterine perforation of IUDs inserted immediately after an abortion. Almost all participants answered correctly the questions about the overall safety of IPP-IUD (100%) and IPA-IUD (98.4%) insertions, and most gave correct answers to the questions about the risks of endometritis in IUD insertions after vaginal births (72.4%), IPA-IUD expulsion rates (63%), and contraindications for IPP-IUD and IPA-IUD use (60.6%) ( Table 2 ).

**Table 2:** Knowledge of 127 on-duty physicians about immediate postpartum and postabortion intrauterine device insertion

Questions	n (%)
1. Is it safe to insert an IUD in the immediate postpartum period?	
Yes (correct)	127 (100)
No	0
2. Is it safe to insert an IUD in the immediate postabortion period?	
Yes (correct)	125 (98.4)
No	2 (1.6)
3. Usual expulsion rate of IUD inserted immediately after a vaginal birth	
> 27%	19 (15.0)
16–26%	51 (40.2)
5–15% (correct)	45 (35.4)
< 5%	12 (9.4)
4. Usual expulsion rate of IUD inserted immediately after a cesarean section	
> 16%	17 (13.4)
11–16%	30 (23.6)
5–10%	51 (40.2)
< 5% (correct)	29 (22.8)
5. Usual expulsion rate of IUD inserted immediately after an abortion	
< 6% (correct)	80 (63.0)
6–11%	29 (22.8)
12–16%	11 (8.7)
> 17%	7 (5.5)
6. Usual risk of endometritis in IUD insertion immediately after vaginal birth	
< 2% (correct)	92 (72.4)
2–3%	18 (14.2)
4–5%	12 (9.5)
> 6%	5 (3.9)
7. Usual risk of uterine perforation from an IUD inserted immediately after an abortion	
0.1–0.2 per 1,000 insertions	64 (50.4)
1–2 for every 1,000 (correct)	54 (42.5)
3 for every 1,000	4 (3.2)
4 for every 1,000	5 (3.9)
8. Usual risk of uterine perforation from an IUD inserted immediately after vaginal birth	
> 4%	3 (2.4)
2–3%	22 (17.3)
0.5–1%	52 (40.9)
< 0.5% (correct)	50 (39.4)
9. Contraindications to inserting an IUD immediately after childbirth or abortion	
Infected abortion and chorioamnionitis (correct)	77 (60.6)
Rupture of membranes for more than 12 hours	33 (26.0)
Infected abortion	1 (0.8)
Chorioamnionitis	0
Women with diabetes	0

Abbreviation: IUD, intrauterine device

Most professionals (73.2%, *n* = 93) would probably or certainly insert an IPP-IUD in themselves or family members, and almost 93% ( *n* = 118) think it is important or very important to increase postpartum IUD use in Brazil. About 72% ( *n* = 91) of the respondents stated that they frequently (i.e., for ≥ 50% of eligible women) recommend IUD use for patients managed in public gynecology outpatient clinics, while 53.5% ( *n* = 68) do so for women managed in private outpatient gynecology clinics. The proportion of physicians who frequently recommend IPP-IUD to eligible women was nearly two times higher for women managed in public than in private hospitals (61.4% versus 29.9%, respectively). Less than half of the participants responded that they frequently recommend the use of IPA-IUD for women managed in public or private hospitals (48.0% and 26.8%, respectively) ( Table 3 ).

**Table 3:** Attitude of 127 on-duty physicians about immediate postpartum and postabortion intrauterine device insertion

Question	n (%)
Would you have an IUD inserted in yourself or a family member immediately after giving birth?	
certainly not	14 (11.1)
probably not	20 (15.7)
probably yes	31 (24.4)
certainly yes	62 (48.8)
Do you think it is important to increase the use of postpartum IUDs in Brazil?	
Very important	92 (72.4)
Important	26 (20.5)
Slightly important	6 (4.7)
Not at all important	3 (2.4)
Recommends IUD use for eligible women in a public gynecology outpatient clinic	
Never	7 (5.5)
Rarely (to < 10% of eligible women)	6 (4.7)
Sometimes (to 10–49% of eligible women)	23 (18.1)
Frequently (to ≥ 50% of eligible women)	91 (71.7)
Recommends IUDs for eligible women in a private gynecology outpatient clinic	
never	14 (11.1)
rarely (to < 10% of eligible women)	12 (9.4)
sometimes (to 10–49% of eligible women)	33 (26.0)
frequently (to ≥ 50% of eligible women)	68 (53.5)
Recommends IPP-IUD use in public hospitals	
never	9 (7.1)
rarely (to < 10% of eligible women)	15 (11.8)
sometimes (to 10–49% of eligible women)	25 (19.7)
frequently (to ≥ 50% of eligible women)	78 (61.4)
Recommends IPP-IUD use in private hospitals	
never	40 (31.5)
rarely (to < 10% of eligible women)	24 (18.9)
sometimes (to 10–49% of eligible women)	25 (19.7)
frequently (to ≥ 50% of eligible women)	38 (29.9)
Recommends IPA-IUD use in public hospitals	
never	17 (13.4)
rarely (to < 10% of eligible women)	24 (18.9)
sometimes (to 10–49% of eligible women)	25 (19.7)
frequently (to ≥ 50% of eligible women)	61 (48.0)
Recommends IPA-IUD use in private hospitals	
never	43 (33.9)
rarely (to < 10% of eligible women)	22 (17.3)
sometimes (to 10–49% of eligible women)	28 (22.0)
frequently (to ≥ 50% of eligible women)	34 (26.8)

Abbreviations: IPA-IUD, immediate postabortion IUD insertion; IPP-IUD, immediate postpartum IUD insertion; IUD, intrauterine device.

About 58% ( *n* = 74) of the participants reported that they had participated in some type of training about IPP-IUD or IPA-IUD insertion. Most of these physicians (74.3%, *n* = 55/74) informed that the training had occurred more than 12 months before, had been promoted by public authorities (Ministry of Health or local Department of Health), and had taken place at the hospital where they worked (59.5%, *n* = 44). Almost 54% of the participants ( *n* = 68) reported that they had not inserted an IPA-IUD in the past 12 months, ∼ 23% ( *n* = 29) had not inserted an IUD immediately after a vaginal birth, and nearly 20% ( *n* = 25) reported that they had not inserted an IUD during a cesarean section in the past year ( Table 4 ).

**Table 4:** Training and experience of 127 on-duty physicians about immediate postpartum and postabortion intrauterine device insertion

Questions	n (%)	
Received training on IPP-IUD or IPA-IUD insertion			
Yes	74 (58.3)	
No	53 (41.7)	
How long ago was this training ( *n* = 74)			
< 12 months	19 (25.7)	
12–24 months	30 (40.5)	
> 24 months	25 (33.8)	
Where did training take place ( *n* = 74)			
At my own hospital, promoted by public health authorities*	44 (59.5)	
During medical residency	34 (45.9)	
At a congress/conference/symposium	15 (20.3)	
Number of IPP-IUD insertions after a vaginal birth the last 12 months ( *n* = 127)			
0	29 (22.8)	
1–5 per month	63 (49.6)	
6–10 per month	19 (15.0)	
> 10 per month	16 (12.6)	
Number of IPP-IUD insertions in cesarean section in the last 12 months ( *n* = 127)			
0	25 (19.7)	
1–5 per month	65 (51.2)	
6–10 per month	20 (15.7)	
> 10 per month	17 (13.4)	
Number of IPA-IUD insertions in the last 12 months ( *n* = 127)			
0	68 (53.5)	
1–5 per month	40 (31.5)	
6–10 per month	11 (8.7)	
> 10 per month	8 (6.3)	

Abbreviations: IPA-IUD, immediate postabortion IUD insertion; IPP-IUD, immediate postpartum IUD insertion.

* Ministry of health or local health department.

Over 70% of the participants consider women's resistance to the method as an important or very important barrier to the insertion of IPP-IUD or IPA-IUD in the public hospital where they work. Over 60% of the participants pointed to the unavailability of copper IUDs in the labor and delivery wards and the lack of hospital guidelines as important or very important barriers to IPP-IUD or IPA-IUD insertion. Other important or very important barriers mentioned by most participants were the lack of experience of the doctors and fear of IUD expulsion (in insertions after vaginal or cesarean deliveries), fear of infection or perforation (in insertions after a vaginal birth or an abortion), and lack of support from hospital managers (for IPA-IUD insertion) ( Table 5 ).

**Table 5:** Barriers to immediate postpartum and postabortion intrauterine device insertion in Brazilian public hospitals

Possible barriers	Postvaginal birth IUD insertion	Postcesarean section IUD insertion	Postabortion IUD insertion
	n (%)	n (%)	n (%)
IUDs are not available in labor/delivery ward			
Not at all important	28 (22.0)	31 (24.4)	34 (26.8)
Somewhat important	18 (14.2)	12 (9.5)	9 (7.0)
Important	32 (25.2)	30 (23.6)	26 (20.5)
Very important	49 (38.6)	54 (42.5)	58 (45.7)
Women's resistance			
Not at all important	9 (7.1)	10 (7.9)	8 (6.3)
Somewhat important	23 (18.1)	25 (19.7)	25 (19.7)
Important	48 (37.8)	53 (41.7)	51 (40.2)
Very important	47 (37.0)	39 (30.7)	43 (33.8)
Hospital does not have guideline for insertion			
Not at all important	27 (21.2)	30 (23.6)	17 (13.4)
Somewhat important	18 (14.2)	17 (13.4)	15 (11.8)
Important	41 (32.3)	42 (33.1)	39 (30.7)
Very important	41 (32.3)	38 (29.9)	56 (44.1)
On-duty physicians lack experience in these insertions			
Not at all important	18 (14.2)	32 (25.2)	14 (11.0)
Somewhat important	24 (18.9)	24 (18.9)	27 (21.3)
Important	48 (37.8)	40 (31.5)	46 (36.2)
Very important	37 (29.1)	31 (24.4)	40 (31.5)
Lack of support from hospital managers			
Not at all important	42 (33.1)	42 (33.1)	36 (28.3)
Somewhat important	25 (19.7)	23 (18.1)	26 (20.5)
Important	26 (20.5)	33 (26.0)	35 (27.6)
Very important	34 (26.7)	29 (22.8)	30 (23.6)
Fear of risk of IUD expulsion			
Not at all important	17 (13.4)	23 (18.1)	23 (18.1)
Somewhat important	29 (22.8)	38 (29.9)	43 (33.8)
Important	50 (39.4)	48 (37.8)	43 (33.8)
Very important	31 (24.4)	18 (14.2)	18 (14.2)
Fear of risk of postinsertion infection			
Not at all important	17 (13.4)	23 (18.1)	13 (10.2)
Somewhat important	34 (26.8)	42 (33.1)	37 (29.1)
Important	53 (41.7)	44 (34.6)	50 (39.4)
Very important	23 (18.1)	18 (14.2)	27 (21.3)
Fear of risk of uterine perforation			
Not at all important	19 (15.0)	43 (33.9)	18 (14.2)
Somewhat important	44 (34.6)	46 (36.2)	45 (35.4)
Important	45 (35.4)	28 (22.0)	42 (33.1)
Very important	19 (15.0)	10 (7.9)	22 (17.3)

Abbreviation: IUD, intrauterine device.

## Discussion

The findings of this national survey indicate that on-duty physicians working in Brazilian public hospitals have limited knowledge about IPP-IUD and IPA-IUD insertion. Most participants have a favorable attitude about IPP-IUD but not about IPA-IUD use. A large percentage of respondents did not have any previous training on IPP-IUD and IPA-IUD insertion and have not performed these types of insertions in the past 12 months. The main barriers pointed out as important or very important for IPP-IUD and IPA-IUD insertions were the resistance of women, the unavailability of IUDs in labor and delivery wards, the lack of hospital guidelines for these insertions, and the lack of experience of the on-duty physicians.

The general knowledge of our participants regarding IPP-IUD and IPA-IUD was similar to that reported in comparable studies involving health professionals from developed countries and better than in studies conducted in low- or middle-income countries. 15 21 22 23 24 Although most Brazilian physicians were aware of the general safety of IPP-IUD and IPA-IUD insertions and their main contraindications, they had some knowledge gaps about the specific risks associated with this type of insertion. For instance, most Brazilian physicians overestimated the risks of expulsion and perforation in IUDs inserted immediately after a vaginal birth. Similarly, authors of a survey involving 58 American physicians working in teaching hospitals reported that less than half gave correct answers to questions about expulsion rates of IUDs inserted immediately after a vaginal birth and perforation rates of IUDs inserted immediately after an abortion. 24 Healthcare providers' overestimation of the risks associated with IPP-IUD use may contribute to the underutilization of the method. 21

The attitude of most of our participants toward IPP-IUD and IPA-IUD insertion was heterogeneous. While most respondents seem to have a positive attitude about IPP-IUD insertion for themselves/family members and in women giving birth in public hospitals, most physicians have less favorable attitudes about IPA-IUD for women managed in the public and private sectors. This could be due participants' lack of knowledge, training, and confidence about IPA-IUD insertion. In agreement with our findings, an American study involving 97 health professionals (32% physicians) working in family-planning clinics reported that 30% of the participants did not believe that IPA-IUD was appropriate and safe. 28 We observed a difference in the attitude of Brazilian physicians when recommending IUD use for women managed in public versus private sectors. In all scenarios (gynecology clinic, immediate postpartum or postabortion), Brazilian physicians recommended IUD insertion to fewer eligible women managed in the private sector than in the public sector. We found no other studies that evaluated the attitude of health providers about IPP-IUD and IPA-IUD use for women managed in different health sectors. It is possible that this attitude may reflect the popular, albeit anecdotal, perception of Brazilian OB-GYNs that the copper IUD is a less sophisticated or modern contraceptive method than the levonorgestrel IUD, a contraceptive method with similar efficacy to that of the copper IUD but that is much more expensive and not available in the public sector. 9

The limited practice of many study participants in IPP-IUD and IPA-IUD insertions may be due to several factors. These include personal issues (lack of confidence, training, or negative attitude toward the method), institutional deficiencies (unavailability of IUDs, lack of hospital support and guidelines), and patient-related factors (lack of knowledge or rejection of the method) identified in the questions about barriers to device use. Women's resistance to the method, one of the main barriers to IPP-IUD and IPA-IUD use according to our respondents, is also reported in the literature and could be due to women's lack of information about the availability of IUDs in delivery wards and lack of knowledge about the contraceptive efficacy of IUDs inserted immediately after birth or abortion. 29 According to a Brazilian study conducted in a public hospital in Campinas, 42% of 242 women refused the offer for free IPP-IUD insertion, and the most important reason was misinformation related to fear of pain, method failure, increased menstrual bleeding, and effects of IUDs on future fertility. 30 Education about the method during prenatal care can significantly increase women's decision to insert IPP-IUD. 31 On the other hand, research indicates that physicians are the greatest source of influence on women's attitude about and choice of contraceptive methods. 32 Considering the impact that physicians have on women's contraceptive decisions, it is important to improve the knowledge, attitude, and practical experience of Brazilian physicians about IPP-IUD and IPA-IUD use to overcome their own resistance to this method. The other three major barriers to IPP-IUD and IPA-IUD insertion in this survey (unavailability of IUDs in the labor and delivery wards, lack of experience of on-duty physicians, and lack of hospital guidelines) are organizational issues that could be solved with relatively simple institutional interventions. The lack of IUDs in labor and delivery wards should not be a barrier to IPP-IUD and IPA-IUD insertions in Brazilian public hospitals because the Ministry of Health has made copper IUDs available to all maternity hospitals in the Unified Health System since 2017. 33 This suggests possible administrative problems, or lack of knowledge of hospital managers, to ensure the continuous and uninterrupted supply of copper IUDs in the labor and delivery wards of all public Brazilian hospitals.

This study has several strengths, including its originality, the use of the best available evidence to design the survey questions and answers, and the pilot testing phase of the questionnaire in a group of volunteers before it was sent to the final participants. This survey is unique in its inclusion of questions to detect possible differences in participants' attitudes toward IPP-IUD and IPA-IUD insertion in patients managed in the public and private sectors, and questions to gather participants' views on the main barriers to the use of this contraceptive method in their own hospitals. The main limitation of this study was that most of the hospitals contacted did not respond to or declined the invitation to participate in the survey. This probably occurred because the study coincided with the first wave of the COVID-19 pandemic in Brazil, when the attention of hospital directors was totally focused on reorganizing their infrastructure and staff to manage this public health emergency. Despite the low adherence of hospitals, we were able to include institutions located in the five Brazilian geographic regions, and the distribution of participating hospitals was proportional to the total number of births in the country. Another limitation of the study was its exclusively quantitative design, involving only close-ended questions. Ideally, the online survey could have included open-ended questions, and we could have complemented the study with individual online interviews or focus groups with a sample of the participants (mixed methods quantitative-qualitative study). This could have allowed a more in-depth analysis of physicians' attitudes about IPP-IUD and IPA-IUD use, and the identification of additional barriers to the use of this contraceptive method. Finally, the findings of this study cannot be generalized to all Brazilian public hospitals, because almost all participating institutions were teaching hospitals and over 20% of the physicians had postgraduate degrees.

The results of this study have several implications for practice. The limited knowledge of the participants about IPP-IUD was surprising since most of them report having received specific training about this type of insertion promoted by public health authorities. This finding indicates the need to reevaluate and improve the quality of the theoretical training currently offered by these authorities or offer refresher courses. To overcome the lack of experience detected in this study, authorities could consider the creation of a practical training module, including hands-on clinical demonstrations and supervision by a tutor in the labor and delivery wards, after the theoretical module. This could increase the experience as well as the confidence of on-duty physicians about IPP-IUD and especially about IPA-IUD insertion in public Brazilian hospitals. The involvement and support of clinical directors and hospital managers are essential to overcome the main organizational barriers to IPP-IUD and IPA-IUD insertion reported by study participants.

This study can serve as a model for similar surveys involving other types of participants (e.g., residents) and institutions (non-teaching, smaller or private hospitals) in Brazil. New studies could also include a qualitative component to further investigate participants' attitudes and identify additional barriers to IPP-IUD and IPA-IUD use. Finally, results suggest the need for studies involving pregnant and postpartum women, as well as women who have just gone through an early pregnancy loss, to investigate their knowledge and attitude about IPP-IUD and IPA-IUD insertion. The results of these new studies will be useful to help develop effective strategies to expand the use of this contraceptive method in Brazil.

## Conclusion

On-duty physicians working in public Brazilian hospitals have a limited knowledge about IPP-IUD and IPA-IUD insertion. Most physicians have a positive attitude toward IPP-IUD insertion, especially for women managed in the public sector, but their attitude is less favorable toward IPA-IUD insertion. A large percentage of participants reported lack of training and experience in IPP-IUD and especially in IPA-IUD insertions. The main barriers to the use of this method in public hospitals are the resistance of women, unavailability of IUDs in the labor and delivery wards, lack of institutional guidelines, and physicians' lack of experience.
